# Perceptions of hospital staff on the performance of Quality Improvement teams in the regional referral hospitals in Tanzania: A cross sectional study

**DOI:** 10.1371/journal.pone.0246927

**Published:** 2021-02-16

**Authors:** Godfrey Kacholi, Ozayr H. Mahomed

**Affiliations:** 1 Discipline of Public Health Medicine, University of KwaZulu-Natal, Durban, South Africa; 2 Department of Health Systems Management, Mzumbe University, Morogoro, Tanzania; BCS Health & Care Scotland, UNITED KINGDOM

## Abstract

**Background:**

To ensure patient-centered quality care for all citizens, Quality Improvement (QI) teams have been established across all public hospitals in Tanzania. However, little is known about how hospital staff perceive the performance of hospital QI teams in Tanzania. This study assessed the perceptions of hospital staff of the performance of QI teams in selected regional referral hospitals in Tanzania.

**Methods:**

This cross-sectional study was conducted in four selected regional referral hospitals between April and August 2018. A self-administered questionnaire was used to collect data from 385 hospital staff in the selected hospitals. Measures of central tendency, proportions and frequencies were used to assess level of perception of hospital staff. Bivariate and multivariate logistic regression was used to test the association between the perceptions of hospital staff of the performance of QI teams and their socio-demographic factors.

**Results:**

The overall mean perception score of the performance of QI teams was 4.84 ± 1.25. Hospital staff aged 35 and over (n = 130; 68%), female hospital staff (n = 144; 64%), staff in clinical units (n = 136; 63%) and staff with post-secondary education (n = 175; 63%) perceived that the performance of QI teams was good. Improved hospital cleanliness was viewed as strength of QI teams, whilst inadequate sharing of information and inadequate reduction in patient waiting time were considered as weaknesses of QI team performance. Bivariate and multivariate logistic regression analyses showed that there was no statistical association between the perceptions of hospital staff and their socio-demographic characteristics.

**Conclusion:**

The overall perception of hospital staff of the performance of QI teams was good, with the main limitation being sharing of hospital QI plans with hospital staff. Hospital staff should be involved in the development and implementation of hospital QI plans, which would promote a positive perception of staff of the performance of QI teams and enhance sustainability of QI teams.

## Introduction

The Tanzania Quality Improvement Framework (TQIF) of 2004 was regarded as the modus operandi for delivering quality healthcare services [1]. The framework was developed to respond to challenges associated with the expansion of health services in Tanzania [2]. In order to promote a high-performance culture of continuous quality improvement (QI) whereby everyone working in the health sector is made responsible for quality, a number of policies and strategies were implemented as a result of the TQIF, resulting in the improved quality of healthcare and health outcomes [3]. Under-5 five mortality rate declined from 147 to 67 deaths per 1000 live births between 1999 and 2016 respectively [4], the rate of attendance at antenatal clinics by pregnant women increased from 43% in 2010 to 51% in 2016 [4], whilst HIV prevalence among adults aged 15 and over declined from 7% in 2004 to 4.9% in 2017 [5].

As a result of the increased demand for quality healthcare services in Tanzania, the TQIF was reviewed and the Tanzania Quality Improvement Framework in Health Care (TQIFH) was launched in 2011 [1]. The updated framework has two main purposes: (1) to ensure that healthcare workers and key stakeholders at all levels in the health sector, design and implement evidence-based innovative approaches for QI; and (2) to identify appropriate actions that should be implemented to institutionalize continuous QI at all levels of healthcare delivery in line with the country’s interests and vision [1]. The updated framework provides strategies to increase clients’ satisfaction at various levels of service provision in the country [3].

To improve the quality of healthcare at all levels of service delivery, the new framework recommended the establishment of Quality Improvement Teams (QI teams) as a concrete structure in health facilities [3]. Unlike the QI teams that were instituted in the former framework, which were mainly established to spearhead an improvement in HIV/AIDS services in their respective facilities, the latter QI teams were established to ensure that health facilities are effective and meet patients’ expectations and coordinate the implementation, management and sustainability of QI interventions at the hospital level [1]. In this study, QI interventions include improving hospital efficiency, reducing unnecessary delays, undertaking internal hospital assessments and improving the appropriateness of hospital use, such as the length of time in hospital and the management of referrals [2, 6].

QI teams play an important advisory role in the health facility planning process, providing input on viable strategies for improving healthcare service delivery, and are considered an important link between the hospital management and staff [1]. The QI teams comprise members from middle to top management in the health facility, and are multidisciplinary, with members from clinical, non-clinical and allied health professions [1]. Each day, QI teams oversee the process of improving the quality of care in the health facilities, including conducting periodic assessments of performance and liaising with the health facility management in implementing the recommended improvement strategies [3]. The close relationship between the QI teams and the hospital management and staff plays a pivotal role in accelerating the QI decision-making process, and increasing the commitment to providing timely and responsive clinical and non-clinical services [1].

A number of studies have shown that QI teams are important for supporting and facilitating the optimal functioning of the health facilities [2, 7, 8]. The results from a qualitative study that interviewed 122 administrators, healthcare providers and staff in 11 hospitals showed that QI teams that were working closely with hospital staff improved provision of quality, safe, accessible, effective and acceptable health care services [6]. Furthermore, QI teams contributed significantly to an improvement in service delivery guidelines, and were positively perceived by hospital managements and staff as pillars of innovation and change [9].

Hospital staff supported by QI teams experienced a decline in the day-to-day challenges of delivering quality healthcare [10]. As a result, the staff’s trust of hospital staff in the QI teams became stronger, which lead to better patient care and ultimately improved health outcomes [11]. Previous studies conducted in Tanzania documented several positive impacts of QI teams in terms of reducing the workload of healthcare professionals, increased patient satisfaction and increased job satisfaction [2, 12]. However, the lack of a shared vision between QI teams and hospital staff may negatively affect staff’s and patients’ safety [13].

The delivery of healthcare services takes place in a multifaceted and rapidly evolving environment, due to demographic, technological and epidemiological changes, and patients’ higher expectations in terms of quality and health outcomes [14]. Hospital staff are key to ensuring the provision of quality healthcare services [15]. QI teams have been formed to provide technical guidance to hospital staff on matters relating to QI. The interaction between QI teams and hospital staff is of the utmost importance to improve hospitals’ performance and health outcomes, and increase patients’ satisfaction [6]. It is therefore indispensable that hospital staff and QI teams work together with a positive attitude and trusting each other [11], which is crucial for achieving the broader goals of improving the quality of care and ultimately the health outcomes of patients [16].

This study builds on several studies that have examined the sustainability of QI teams [2], the contribution of QI teams to healthcare delivery [12, 17] and association between the healthcare context and the policies governing QI, in terms of communicating and coordinating them, clarifying roles and responsibilities, leadership and management support, and human and financial resources [2, 6–8, 11–13, 16–18]. Although these previous studies examined the general performance of QI teams, there is a paucity of information about the specific activities performed by them and how hospital staff perceive QI teams.

This study sought to assess the perceptions of staff at regional referral hospitals of the performance of QI teams. This study was carried out as a part of a larger study aimed at assessing the sustainability of QI teams in selected regional referral hospitals in Tanzania [2].

## Materials and methods

### Study design and setting

This analytical cross-sectional study was part of a larger quantitative study conducted between April and August 2018. The study was conducted in four selected public regional referral hospitals in four regions in Tanzania. These hospitals are at the secondary level of healthcare provision, serving as the last referral point at the regional level offering more specialized services.

The selected hospitals were Singida Regional Referral hospital in Singida region in central Tanzania; Tanga Regional Referral hospital in Tanga region on the east coast; Mbeya Regional Referral hospital in the southwest highlands; and Sekou-Toure Regional Referral hospital in Mwanza on the shore of Lake Victoria. At the time of the study, some of the key hospital characteristics are indicated in Table 1.

**Table 1:** Profile of the study settings as of 2017.

Regions	Name of the hospital	Regional population in Millions	Bed capacity	Total number of staff	Members of QI teams	Number of departments	Number of sections
Tanga	Tanga RRH	2.05	412	367	22	15	38
Mwanza	Sekou-Toure RRH	2.77	315	362	25	6	9
Singida	Singida RRH	1.37	275	375	14	9	11
Mbeya	Mbeya RRH	2.71	170	312	15	9	24

Source: Ministry of Health and Social Welfare and JICA [19].

### Sampling and sample size

A sample size of 385 was calculated using Yamane’s formula: n = N/1+N (e) ^2 [20], with a 5% margin of error and a 95% confidence interval. Out of the 28 public regional referral hospitals in Tanzania, four hospitals were sampled for this study. A hospital was included if it was in the category of either a high performing or low performing hospital with regard to the progress of implementing QI recorded in the external hospital performance assessment report for regional referral hospitals in 2016 [19].

Grouping high and low performing hospital with regard to QI implementation progress was based on the criteria of External Hospital Performance Assessment (EHPA) for Regional Referral Hospitals as defined in the Guideline for Internal Supportive Supervision (ISS) and External Hospital Performance Assessment (EHPA) for Regional Referral Hospitals [20]. The hospital performance scores are 70% and over (high performing hospital); 41% to 69% (moderate performing hospitals); and under 40% (low performing hospitals). Our interest was to include high and low performing hospitals in this study [21].

The simple random sampling technique was used to select the four regional referral hospitals. Tanga and Singida regional referral hospitals were sampled from the list of high performing hospitals, whilst Sekou-Toure and Mbeya regional referral hospitals were sampled from the list of seven hospitals with low performance scores. From each hospital, all departments were selected for this study, and from each department, three hospital units were randomly selected. From the sampled hospital units, purposive sampling technique was employed to select the participants (hospital staff).

### Data collection tool and procedure

A self-administered questionnaire was used to collect data from hospital staff in the selected hospitals (S1 File), which was in two parts. The first part captured the socio-demographic characteristics of the participants, while the second part assessed the perceptions of staff of the performance of the QI teams.

The questionnaire was prepared in English and translated into Kiswahili (the language spoken by more than 90% of Tanzanians). The Kiswahili version was then back-translated into English to retain the accuracy and consistency of the questionnaire. Two language experts were engaged to validate the accuracy of translation, while three people who have worked in the QI teams for the past four years reviewed the relevance and clarity of the questions. The questionnaire was pre-tested with 25 hospital staff, the results of which are not included in the overall analysis. During data collection, questionnaires were distributed and collected from the study participants at their respective points of service by the principal investigator.

### Variables and measurements

#### Dependent variable

The dependent variable was the perception of staff of the performance of the QI team. Eight items were used to assess this, namely, training, staff involvement, QI team support, staff engagement, patient waiting time, hospital cleanliness, sharing of QI plans and overall satisfaction. For each item, a closed question with a Yes or No response was used to capture the perceptions of the study participants (S1_File).

A composite perception score was obtained after summation of the self-reported items. The maximum score attainable was 8. The mean and median scores were calculated from the composite perception score. Items with a mean score of over 4 were considered to represent the good perception of the study participants, while items with a mean score of under 4 were considered to represent their poor perception of the performance of QI teams. The internal reliability of eight items used to assess the perception of staff of team performance was measured using Cronbach’s alpha (α  =  0.73).

#### Independent variables

Independent variables included socio-demographic characteristics of the study participants: gender (male or female); age (less than 35 or 35 and over); educational level (secondary/less or post-secondary education); professional category (clinical services such as obstetrics and gynecology, pediatrics, oral health, optometry, dermatology, and TB and leprosy) or non-clinical services (such as administration, medical records, social welfare, health information management and nutrition); and length of service (less than or more than 10 years).

### Data analysis

Data was extracted from the questionnaires and then captured using Microsoft Excel, after which it was exported to IBM Statistical Package for Social Sciences (SPSS) version 16.0 in order to analyse the data. Descriptive statistics were used to determine frequencies and proportions for categorical variables, while measures of central tendency were used for continuous data. Bivariate and multivariate logistic regression was used to test for associations between the dependent and independent variables. Odds ratios (OR) with 95% Confidence Intervals (CI) were computed and used to determine the strength of association. Statistical significance was considered for *p* ≤0.05.

### Ethical considerations

This study was approved by the National Institute for Medical Research of Tanzania (NIMR/HQ/R.8a/Vol.IX/2666) and the Biomedical Research and Ethics Committee of the University of KwaZulu-Natal, South Africa (BE: 606/17). The relevant regions and hospital authorities provided gatekeeper to access study participants. Signed written informed consent was obtained from each participant after explaining the purpose, benefits and risks of the study, voluntary nature of participation and that they were free to drop out at any time without giving a reason.

## Results

### Socio-demographic characteristics of study population

Three hundred and eighty five hospital staff were recruited, with 314 (82%) consenting to participate in this study. Seventy one percent (n = 224) of the hospital staff were female, with 61% (n = 192) aged 35 and over, and 88% (n = 276) having completed post-secondary education, and 68% (n = 215) working in the clinical services department. Most hospital staff (66%; n = 208) who took part in this study had worked in their hospital for less than 10 years (Table 2).

**Table 2:** The socio-demographic characteristics of study participants (n = 314).

Socio-demographic characteristics	Frequency	Percent
**Gender**		
Male	90	29
Female	224	71
**Age (in years)** Mean (± SD) 39.9 ±10.4		
≤ 35	122	39
>35	192	61
**Educational level**		
Secondary education	38	12
Post-secondary education	276	88
**Profession category**		
Clinical services	215	68
Non-clinical services	99	32
**Length of service (in years)** Mean (± SD) 10.7± 9.4		
≤ 10	208	66
>10	106	35

### Perceived performance of Quality Improvement teams

**Fig 1:**
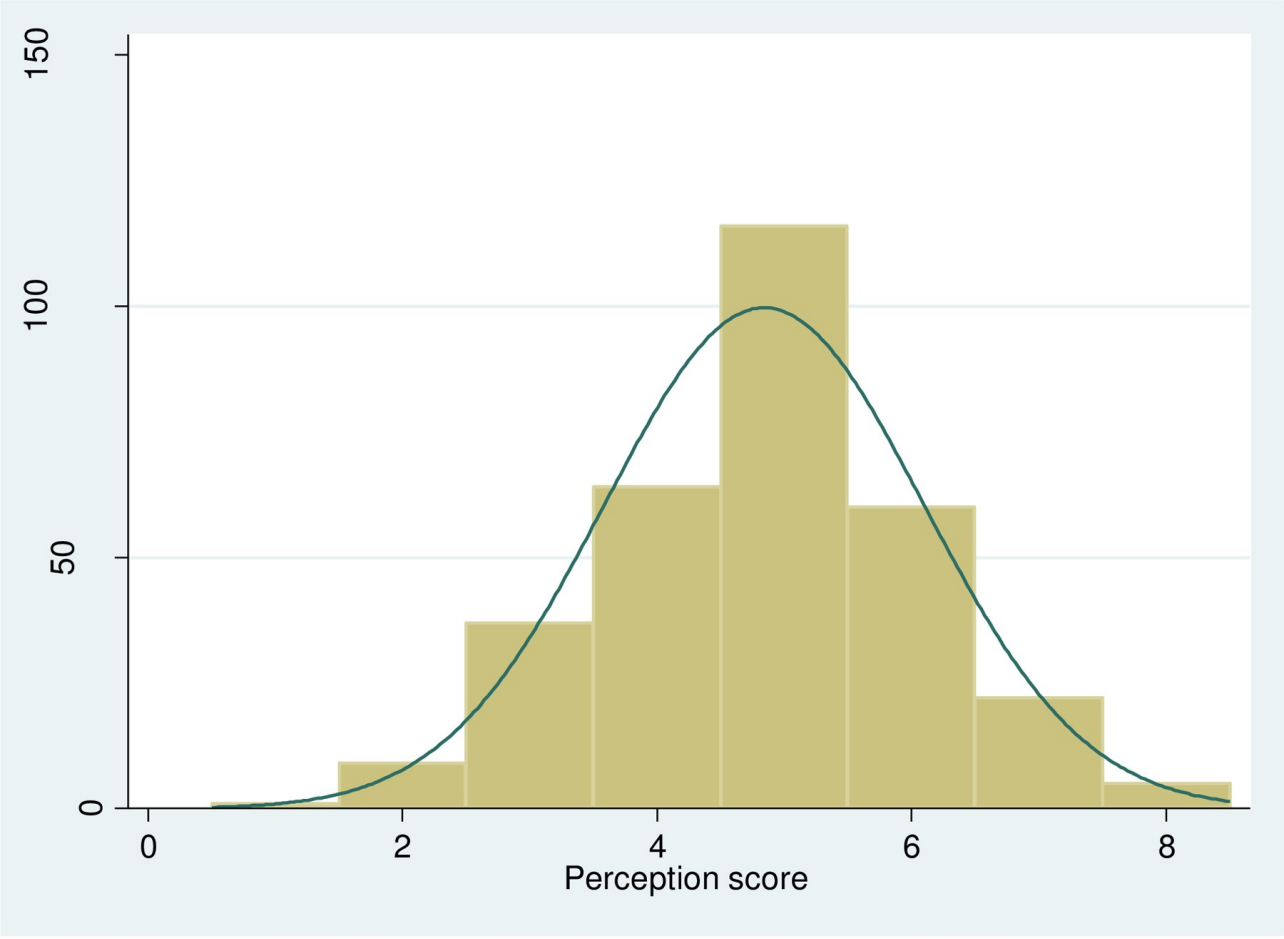
Perception score on the performance of QI teams.

The overall mean perception score of the performance of the QI teams was 4.84 (SD: 1.25).The data was centrally located with the median score of 5 (IQR = 2) (Fig 1).

Female hospital staff (*n* = 144, 64%), hospital staff aged over 35 (n = 130; 68%), with less than 10 years’ length of service (n = 129; 62%), working in clinical units (n = 136; 63%) and with post-secondary education (n = 175; 63%) perceived that the performance of QI teams was good (Table 3).

**Table 3:** Perceptions based on socio-demographic characteristics of staff (n = 314).

Socio-demographic characteristics	Poor Perception < 4		Good Perception ≥ 4	
	Number	Percentage	Number	Percentage
**Gender**				
Male	31	34%	59	66%
Female	80	36%	144	64%
**Age (in years)**				
> 35	62	32%	130	68%
≤35	49	40%	73	60%
**Level of education**				
Post-secondary	101	37%	175	63%
Secondary or less	10	26%	28	74%
**Profession category**				
Clinical services	79	37%	136	63%
Non-clinical services	32	32%	67	68%
**Length of service**				
< 10 years	79	38%	129	62%
> 10 years	32	30%	74	70%

### Perceived performance of QI teams based on socio-demographic characteristics of hospital staff

#### Perceived strengths of the QI teams’ performance

The majority of female staff (n = 168; 75%), l staff aged 35 and over (n = 142; 74%), those working in clinical service units (n = 155; 72%), those with post-secondary education (n = 191; 69%) and those with less than 10 years’ service (n = 139; 67%) perceived that QI teams contributed to overall improved hospital cleanliness. In addition, female staff (n = 159; 71%), hospital staff working in clinical service units (n = 149; 69%), those with post-secondary education (n = 189; 68%) and those aged 35 and over (n = 131; 68%) reported that QI teams conducted QI training courses effectively in the hospitals (Table 4).

**Table 4:** Perceptions of the performance of QI teams based on socio-demographic characteristics of hospital staff (n = 314).

Perceptions	Gender			Age (in years)		Level of education		Profession category		Length of service (in years)	
	Female n (%) N = 224	Male n (%) N = 90	Total n (%) N = 314	≤35 n (%) N = 122	>35 n (%) N = 192	Secondary n (%), N = 38	Post-secondary n (%), N = 276	Clinical n (%) N = 215	Non-clinical n (%), N = 99	< 10 years (%), N = 208	>10 n (%) N = 106
Team improved hospital cleanliness	168 (75)	54 (60)	222 (71)	80 (66)	142 (74)	31 (82)	191 (69)	155 (72)	67 (68)	139 (67)	83 (78)
Team conducts QI training courses effectively	159 (71)	57 (63)	216 (69)	85 (70)	131 (68)	27 (71)	189 (68)	149 (69)	67 68)	137 (66)	79 (75)
QI plans not shared adequately with staff	137 (61)	68 (70)	205 (65)	87 (71)	118 (61)	23 (61)	182 (66)	138 (64)	67 (68)	146 (70)	59 (56)
Team is supportive of staff when needed	141 (63)	60 (63)	201 (64)	72 (59)	129 (67)	29 (76)	172 (62)	136 (63)	65 (66)	129 (62)	72 (68)
Staff feel inadequately involved in QI implementation	125 (56)	56 (62)	181 (58)	76 (62)	105 (55)	20 (53)	161 (58)	119 (55)	62 (63)	129 (62)	52 (49)
Staff are rarely involved in conducting internal assessments	118 (53)	52 (58)	170 (54)	64 (54)	106 (55)	22 (58)	148 (54)	113 (53)	57 (58)	112 (54)	58 (55)
Long patient waiting time is still a challenge	127(57)	56(62)	183 (58)	75 (61)	108 (56)	22 (58)	161 (58)	123 (57)	60 (61)	130 (63)	53 (50)
Team performance is satisfactory	139 (62)	55 (61)	194 (62)	69 (57)	125 (65)	24 (63)	170 (62)	128 (60)	66 (67)	126 (61)	68 (64)

#### Perceived weaknesses of the QI teams’ performance

Hospital staff with less than 10 years’ service (n = 146; 70%), those with post-secondary education (n = 182; 66%), those working in clinical service units (n = 138; 64%), female staff (n = 137, 61%) and hospital staff aged 35 and over (n = 118; 61%) perceived that QI teams were not sharing hospital QI plans adequately with hospital staff. Furthermore, hospital staff with less than 10 years’ service (n = 130, 63%, those with post-secondary education (n = 161; 58%), female staff (n = 127; 57%) and hospital staff aged 35 and over (n = 108; 56%) indicated that QI teams did not adequately contribute to a reduction in patient waiting time (Table 4).

### Associations of perceptions of hospital staff and their socio-demographic characteristics

Although both bivariate and multivariate logistic regression analyses showed that there was no statistical association between the socio-demographic characteristics of hospital staff and perceptions towards QI teams, both bivariate and multivariate analysis showed an increased odds of a positive perception for staff aged 35 and over (OR 1.22; CI 0.69–2.16; *p* = 0.49). However, males staff, staff with post-secondary education, staff working in clinical services and those with less than 10 years of service showed a decrease in the odds of a positive perception of QI team performance (Table 5).

**Table 5:** Association of perceptions of hospital staff and their socio-demographic factors (n = 314).

Factor	Categories	Unadjusted OR	95% CI	p—value	Adjusted OR	95% CI	p—value
Gender	Male versus female	0.94	0.54–1.62	0.83	0.89	0.51–1.54	0.69
Age (in years)	> 35 versus ≤35	1.40	0.85–2.31	0.15	1.22	0.69–2.16	0.49
Level of Education	Post-secondary versus secondary and less	0.61	0.25–1.38	0.21	0.61	0.27–1.31	0.20
Profession category	Clinical services versus non-clinical services	0.82	0.47–1.39	0.44	0.76	0.44–1.31	0.34
Length of service (in years)	< 10 years versus > 10 years	0.70	0.41–1.19	0.17	0.74	0.40–1.31	0.36

Level of statistical significance *p*<0.05.

## Discussion

Hospital staff interact constantly and directly with QI teams while carrying out day-to-day hospital activities. Therefore, the perceptions and opinions of hospital staff on the performance of QI teams are fundamental for improving the quality of healthcare services. To our knowledge and from the literature search, this study is one of the first to assess the perceptions of hospital staff of the performance of QI teams in regional referral hospitals in Tanzania [1–7, 12, 13, 20].

Our study indicates that hospital staff had a positive perception of QI teams’ performance. Similar findings were noted in a mixed-methods study conducted in two mission referral hospitals in Kenya, which found that nurses had a good perception of the hospital QI teams [22]. In another study conducted in New Zealand, health professionals had confidence with QI teams in improving patients’ health outcomes [23]. This could be because of the contribution made by QI teams in improving quality of health services [2].

The present study showed that most hospital staff saw that the QI teams made a contribution through improved hospital cleanliness and conducting effective QI training courses. QI teams used in-house QI-related training to sensitize staff to develop a culture of cleanliness to improve the environment so as to provide safe and effective care. Improved hospital cleanliness reduces the number of infections associated with hospitals, and can greatly raise staff’s morale and increase job satisfaction [24]. Continuous training of healthcare workers reinforces and sustains the change process whilst simultaneously improving the overall performance of health facilities [25].

Both males and females appeared to have a positive perception of the performance of QI teams, although the majority of hospital staff who participated in this study were female, which may be due to the fact that the majority of healthcare staff in Tanzania are female [26]. These results are in line with the results of a qualitative study conducted at a district hospital in KwaZulu-Natal, which found that the majority (95%) of study participants were female [18]. Although this study did not establish a significant association between gender and perception of the performance of QI teams, the results from a cross-sectional study conducted at Stanger Hospital in South Africa found that the majority of the staff were female, and therefore gender composition could influence the perception of staff [27].

This study’s participants felt that hospital QI plans were not shared adequately with hospital staff, maybe because QI teams do not see the value of involving hospital staff in the QI planning process or the contribution they make to improve the provision of healthcare services, which may lead to hospital staff feeling isolated and underrated by the QI teams. Extrapolating data obtained about the perceptions of healthcare workers of the organisational quality assurance intervention implemented in resource-limited contexts, it has been hypothesized that healthcare workers tend to have a positive perception of newly introduced interventions when they are involved in the planning, implementation, monitoring and evaluation stages [28]. From the sustainability point of view, the involvement of hospital staff in the planning process and developing hospital QI plans increases their morale, improves productivity and binds them together [29].

This study found that the professionalism (clinical versus non-clinical) of hospital staff was not statistically associated with their perception of the performance of QI teams. However, hospital staff working in clinical service units appeared to be impressed by the performance of QI teams. This result is consistent with the findings of a descriptive correlational study conducted in Ontario, which found that health professionals (nurses) had a positive attitude towards the performance of inter-professional healthcare teams [30]. Despite the differences between the perceptions of clinical and non-clinical support staff of the performance of QI teams, the interaction between health professionals and QI teams has been essential for ensuring that QI teams remain active and functional [6].

Although no statistical association between the age of hospital staff and their perception of the performance of QI teams, the results showed that older staff appeared to be impressed by the performance of QI teams. These findings are in line with those of a cross-sectional study conducted in 42 health facilities in South Africa, which showed that older staff had a positive perception of quality improvement interventions [14]. Contrary to these results, a previous study observed that younger staff were less impressed with the performance of the QI team [31], which may be due to younger staff being more ambitious and opinionated than older staff. Some studies have shown that younger employees in the workplace are more innovative, faster learners, more energetic and possess more knowledge of new technology [32]. This implies that the effect of age on perceptions is variable.

This study did not find any significant association between the level of education of hospital staff (post-secondary education) and their perception of the performance of QI teams. The level of education of health professionals has an influence on their perception of the interventions introduced and implemented in their respective health facilities [33, 34]. Staff with a high level of education are more likely to have a positive perception than less educated staff [33]. This may be attributed to the amount of knowledge acquired through informal and informal training, which enables hospital staff to become more informed about the changes taking place in their health facilities and within the health sector [35].

### Study limitations

Although due diligence was observed in undertaking this study, the results should be interpreted in the light of the following limitations. Firstly, the dimensions used to assess the perceptions of staff of the performance QI teams were not fully exhausted. Secondly, we did not collect qualitative data that could be useful for providing in-depth information on issues captured in the quantitative results. Thirdly, the study participants were conveniently selected, which could have led to selection bias.

### Conclusion and recommendations

The hospital staff’s perception of the performance of QI teams in the surveyed regional referral hospitals was good, with contribution towards improved hospital cleanliness and the effective QI training courses organised for hospital staff considered as strengths. Little or no effect in reducing patients’ waiting time and QI teams not sharing hospital QI plans with hospital staff appeared to be the main weaknesses of these teams. There was no statistical association between the perception of hospital staff and their socio-demographic characteristics. A further qualitative study involving hospital and QI teams is required to have a deeper understanding of impression and challenges experienced with respect to QI teams. As a measure to improve the perception of QI team and to give hospital staff a sense of ownership and ensure sustainability of QI teams efforts, it is recommended that hospital staff should be involved in the development and implementation of hospital QI plans, which would promote a positive perception of staff on the performance of QI teams and enhance sustainability of QI teams.

## Supporting information

S1 Data (XLSX)

S1 File (DOCX)
