# Supplementary material for: Perceptions of hospital staff on the performance of Quality Improvement teams in the regional referral hospitals in Tanzania: A cross sectional study
Source: PLoS One. 2021 Feb 16;16(2):e0246927. doi: 10.1371/journal.pone.0246927 (PMC7886151; doi:10.1371/journal.pone.0246927)
Supplement: S1 File — (DOCX) [file pone.0246927.s002.docx]

**Survey Tool for Hospital Staff**

Dear Sir/Madam

This study aims to assess the perceptions of hospital staff on the performance of Quality Improvement Teams in the selected Regional Referral hospitals in Tanzania. Your participation in this study is highly respected.

Name of the hospital: __________________________________

Name of the region: ____________________________________

Date (day/month/year):_________________________________

**Information of Respondents**

Please circle for a relevant and an appropriate answer

1. Gender
2. Male
3. Female
4. What is your age (in years?) ____________________________
5. What is your level of education?
6. Secondary education
7. Post-secondary education
8. In which professional category do you belong?
9. Clinical services
10. Non-clinical services
11. How long have you been working in this hospital? (years) __________________

| **Items** | **Yes** | **No** |
| --- | --- | --- |
| 1. Team improved the overall hospital cleanliness |  |  |
| 1. Team conducts QI trainings effectively |  |  |
| 1. QI plans inadequately shared with staff |  |  |
| 1. Team is supportive to staff when needed |  |  |
| 1. Staff feels inadequately involved in QI implementation |  |  |
| 1. Staff are less involved in conducting internal assessments |  |  |
| 1. Long patient waiting times is still a challenge |  |  |
| 1. Team performance is satisfactory |  |  |

**Thanks for Your Participation**
